# Investigating preferences for support with life after stroke: a discrete choice experiment

**DOI:** 10.1186/1472-6963-14-63

**Published:** 2014-02-08

**Authors:** Christopher R Burton, Emily Fargher, Catrin Plumpton, Gwerfyl W Roberts, Heledd Owen, Eryl Roberts

**Affiliations:** 1 School of Healthcare Sciences, College of Health and Behavioural Sciences, Bangor University, Bangor, Gwynedd LL57 2EF, UK; 2 Centre for Economics and Policy in Health, Bangor University, Bangor, UK; 3 National Institute for Social Care and Health Research Clinical Research Centre, Bangor, UK

## Abstract

**Background:**

There is little evidence of service user preferences to guide the commissioning and improvement of services that support life after stroke. We report the first investigation of patients’ and family carers’ preferences for community services after stroke using a discrete choice experiment (DCE).

**Methods:**

Two workshops with patients and family carers (n = 8) explored stroke experiences, identifying attributes important in shaping views about service design, and piloted data collection strategies. Attributes were group versus individual support; service provider; additional support for social and leisure activities; and the total time required to access services. Patients and family carers were recruited six months post stroke-onset (mean 331 days) from four stroke services, and invited to participate in the DCE. Patients’ general health (EQ5D) and functional dependence (Barthel Index) were also assessed. Of 474 eligible patients, 144 (30%) expressed an interest in the study, and 80 (56%) of these completed the survey questionnaire. 34 of 74 (46%) family carers recruited through patients completed the DCE.

**Results:**

All four attributes were significant in shaping patients preferences for stroke support service delivery (p < 0.05), confirming the interpretation of workshop findings. Patients prefer help and support for emotional needs, communication problems and physical difficulties to be provided on an individual basis; and to be offered additional social and leisure activities that they are able to attend on their own. Patients would appear to prefer that voluntary organisations do not provide these services, although this may be linked to lack of experience of these services. Family carers would prefer help and support in their caring role on a one-to-one basis. Whilst health related quality of life is associated with preference for format of service, results were relatively consistent across sub-groups, with the exception of time since stroke, where social and leisure activities had a greater impact on preferences of established service users.

**Conclusions:**

The data provide unique insights into how preferences for community services that support life after stroke are shaped. This information can be used to inform both service re-design, and barriers to implementation that will need to be accounted for in policy shifts towards a more mixed economy of service provision.

## Background

According to the World Health Organization, stroke is one of the leading global causes of death and disability [1]. With an annual incidence of 110,000 in England, stroke is the third most common cause of death representing expenditure in health and social care in the region of £7 billion a year [2]. This figure reflects the complex picture of physical, emotional and social stroke-related sequelae that can have far-reaching consequences for patients and families. The international evidence-based stroke service model spans prevention interventions in primary care; inpatient hyper-acute and acute care; rehabilitation; on-going community support; and palliative care (e.g. [3-6]). Health policy recognises that the personal, family and social impacts of stroke can be long-term, and persist long after early rehabilitation services are withdrawn (e.g. [7,8]). Consequently, a complex matrix of health and social care services from across the statutory, voluntary and independent sectors, may be required to support life after stroke. Inevitably, there is substantial variation in how these needs are met at a local level, with limited information of the relative effectiveness or acceptability of alternative service models. To our knowledge, this paper reports the first investigation of patient and family carer preferences for services that deliver on-going community support for life after stroke.

It is increasingly acknowledged that recovery can continue for some time after stroke [9], and patients and family carers should have access to resources that facilitate on-going review and rehabilitation. Specialist teams may be more important in the early stages of rehabilitation, while generic teams, with the appropriate knowledge and skills, can be beneficial later on [8]. Many people experience emotional difficulties after stroke that can impact on physical recovery, mental well-being and social isolation [10]. In addition, carers are vulnerable to problems relating to coping and depression [11].

The evidence of patient and family carer needs on which to base the design of community services is generally limited to reviews of experience and observational studies which have variable and wide-ranging measurements, many of which are non-standardised. The long-term problems experienced by stroke sufferers and their family carers have been subject to qualitative synthesis [12]. In parallel to this, a systematic review of survey studies reporting prevalence and typology of longer term problems has also been conducted [13]. These reviews identify a range of problem categories, including social and emotional consequences which were the largest domains identified, and which appear to persist many years after stroke [14]. More recently, the UK Stroke Association commissioned a needs survey of people who had been living with stroke for between one and five years [15]. Based on 1252 survey responses, just under one half of all respondents reported that they had unmet needs, indicating that either problems had not resolved, or were not being managed through coping mechanisms.

Within the UK, there is an increasing aspiration for the design of public services that are user-centred, rather than driven solely by professional perspectives [16-18]. This aspiration is embedded within the National Clinical Guidelines [5] which make two recommendations relevant to patient and public involvement:

• The views of stroke patients and their carers should be considered when evaluating a service; one method that should be used is to ask about their experience and which specific aspects need improvement.

• The planning process for any service development should include active involvement of stroke patients and carers, with particular consideration of the views of patient who are unable to participate in the planning process directly.

These recommendations mirror a quality marker (Quality Marker 4) from the English National Stroke Strategy [8] outlining the meaningful involvement of patients and family carers in the planning, development, delivery and monitoring of services.

Summaries of evidence and policy highlight the need for an integrated, flexible whole-system approach to commissioning, planning and developing services that support life after stroke. This should draw on the full range of services (statutory, voluntary and independent), and provide flexibility to address patient and family carer needs over time. As a lack of clear direction is provided by the evidence base, together with significant variations in the availability of community services and resources, and the maturity of partnership working arrangements, mean that there is scope for flexibility in implementation. This flexibility may relate to who provides services, their content, and how they are provided. As the basis for the on-going development of community-based services for people living with stroke, this study will investigate patient and family carer preferences for stroke services and potential trade-offs between various factors associated with service design, using a discrete choice experiment (DCE).

DCE’s are growing in popularity within healthcare [19], providing information on the importance of characteristics (attributes) of services that have currency with service stakeholders, the trade-offs that stakeholders are prepared to make between these characteristics, and the relative importance of these characteristics [20]. To our knowledge, only two other DCE’s have addressed the preferences of stroke patients, investigating the content of early rehabilitation [21] and the role of information in influencing preferences around treatment for ankle and foot impairments [22]. This study will identify the role of actionable factors that significantly influence preferences and could inform a user centred service.

## Methods

This two-phase study investigated patient and family carer preferences for the design of community services to support life after stroke, and trade-offs between service design factors. In Phase One, we identified relevant attributes for the design of health and social care services for people living with stroke, and select appropriate degrees or levels for these attributes. Phase Two was a discrete choice experiment to elicit preferences amongst these attributes. The study was granted NHS research ethics approval on 22nd April 2011 (REC Ref 11/WA/0061), and research governance approval was gained from all participating NHS sites.

We used Coast and Horrocks [23] guidance on the selection of attributes and levels for use in the DCE to ensure their relevance and plausibility within the service context. The study design required consideration of the specific communication needs of potential participants, both in terms of undertaking research within a bilingual context, and in enabling participation of those people with stroke-related communication challenges*.* Communication challenges after stroke can be complex and ‘hidden’ [24]. To address this, a stroke specialist speech and language therapist reviewed all study material to ensure that these were ‘aphasia friendly’. Group interviews with patients and family carers to identify potential attributes for the discrete choice experiment survey included ‘cognitive interview’ questions (e.g. what does this symbol mean to you?) to check the interpretation of symbols and signs used to support textual elements within the DCE.

### Phase one

Workshops were conducted in North West Wales with patients and carers attending peer support groups, each facilitated by two members of the research team. Given the bilingual context of North Wales [25], participants were invited to attend a Welsh or English medium event, according to their language needs or preference. Workshop activities were structured to develop an understanding of the target population’s perspective and experience [23,26]; and relevant health care policy [27]. Workshop participants were invited to:

• Expand on a list of potential attributes identified from a review of health policy [7,8], reports of service user needs and experiences [28], and evidence-based guidance for community services [29].

• Identify the potential range of variation in each attribute.

• Review and refine the final attribute and level selection.

Attention was paid to potential attributes where evidence was missing, or where there were potential variations in delivery and organisation. Mandatory, routinely provided, clinical services were excluded. Engagement in workshop activities was maximised through the use of cards to sort and discuss attributes, with pictures representing issues under discussion. Workshops were co-facilitated by researchers familiar with conducting interview-based research with this study population, specifically in working with people with post-stroke communication challenges. The workshop discussions incorporated a number of choice-making activities where the intended format for seeking preferences, and the selection of aphasia-friendly symbols, could be checked by participants. The workshops were digitally recorded, fully transcribed and analysed in their original language using Framework Analysis [30]. The analytical task across the workshop data was to identify a potential range of attributes that resonated with the experiences of patients and family carers, and to identify dimensions for each attribute. An initial framework of thematic categories was applied to workshop transcripts. New categories were identified during the coding process, where attention was paid to achieving a balance between data relating to first-hand experience of life with stroke, and general attitudinal expressions not grounded in such experience. Coded data were tabulated, with exemplar quotations, to identify different dimensions associated with each category which are summarized in Table 1.

**Table 1:** Overview of categories identified by workshop participants

**Category**	**Dimensions**
Travel to services	Availability of transport; accessibility of home; ease of transport mode; convenience; personal meaning (e.g. ambulances and illness); journey comfort; **journey time**
Adaptations to home	Waiting time
Therapy and support	Pro-active/reactive; timing; emotional content; variability; continuity
Information	Mode; source; timing
Service delivery	Peer support; **one to one support/group support**; family engagement; physical access to services
Family support	Patients as family carers; differences in the purpose of family support
Social aspects	**Leisure activities/work support**
Follow-up assessment	Prior experience of services; **service provider**; service proximity; responsiveness
*Language sensitivity*	Choice; ease when concordant; understanding; emergency contexts; therapeutic elements

Categories in bold taken forward for consideration of levels, and use in DCE.

For further detail of attribute and dimensions, together with exemplar quotations from workshops, please see Additional file 1.

Workshop participants identified a range of dimensions that characterised the challenges of accessing services, including convenience, ease, availability, comfort, and the personal meaning associated with different forms of transport. A transferable attribute around time spanned these dimensions, including the total time taken to prepare for and complete travel associated with accessing services: “*Quickness in time as well. The train is probably quicker but by the time you have got to the station and everything; got here from the station the car*…” (Family carer). Inevitably workshop data demonstrated a wide range of stroke-related problems and needs, many of which were specific to individual circumstances. Although it did not make sense to include specific services as attributes, two potential attributes were identified where trade-offs made sense: who provided services, and the format of delivery.

Workshop participants identified sources of support that spanned hospital, primary care, voluntary and other (e.g. vocational) settings, although how these were accessed appeared to be un-coordinated, for example: “*they said that things would start within 2–3 days of being home, but after 3 months, I didn’t see anyone or get through to anybody. Then I got … an appointment at the speech therapy department, and through them I went to the art workshop*” (Patient). Importantly, these data identified a number of gaps in availability, prompting some to postulate support that might have been helpful: *You would think that there was someone in between [hospital] and [GP]. Someone there in the middle that you could talk to. Sometimes just having someone to listen to your worries*” (Patient). Participants recognised that the availability of some sources and types of support was limited, and discussed attitudes about accessing services individually or in groups:

“even *one to one first [for support] and to be asked if you would like to join a group*”. (Patient)

*“I’m not comfortable in a group really, not that there’s anything wrong with a group, but I don’t feel comfortable”.* (Patient)

Some participants had accessed services and support around social and leisure activities, but this was seen as distinct from more health service oriented programmes:

“*It [leisure activity workshop] didn’t concentrate on any medical things which was nice*”. (Patient)

“*Now that he has got this far what we feel is we could use something like they have at [place] where they have the workshops, the afternoon workshops for people after they have had strokes, they do woodwork and mechanics*”. (Family carer)

Where patients had accessed these types of support, they also provided space for family carers: “*the stroke group gave him two hours off from me, with other people and me two hours off from him to go and root around shops on my own without [patient] being there*” (Family carer). Further detail of the workshop findings are provided in Additional file 1.

### Phase two

The DCE was contained within a bilingual questionnaire-based survey of people living with stroke, with separate questionnaires developed for stroke sufferers and family carer(s). The surveys were conducted in North Wales comprising a mix of rural, urban and industrial settings. Healthcare services for stroke in North Wales comprise three district general hospitals, each of which delivers an acute stroke service, inpatient rehabilitation, and varying ranges of community services such as intermediate care, continuing care, and independent care home services.

### Subjects and sampling

#### ***Patients and family carers***

Adult stroke patients surviving to six months were prospectively identified from stroke registers by NISCHR CRC staff at each of the three stroke services in North Wales. Patients with a diagnosis of stroke between July 2011 and May 2012, and their family carers, were considered for inclusion in the study. Patients admitted to a mid-Wales stroke service between October 2011 and June 2012 and their family carers, were also included in the study using the same study procedures.

#### ***Sample size***

Based on published DCEs in primary care [31], and our experience of recruiting stroke patients for surveys or interviews, we estimated our potential sample size to be 450 patients and/or carers (assuming a response rate of 35% of an estimated 1300 stroke patients surviving to 6 months, in North Wales).

#### ***Data collection***

Data were collected by bilingual postal questionnaire which gathered a minimum of demographic data: marital status, housing arrangements and language background. Details of respondents’ stroke histories were extracted from the hospital records once a questionnaire had been returned. The major, first section of the questionnaire was constructed as a series of nine paired scenarios, each requiring respondents to select one of two preference options. The remaining components of the questionnaires were as follows:

For patients

•An evaluation of general health - EQ5D (including visual analogue scale of health perception at time of study participation) [32,33]

•An evaluation of dependence in activities of living - Barthel Index [34]

For family carers

•An evaluation of family carers’ general health – EQ5D (including visual analogue scale of health perception at time of study participation) [32,33]

•An evaluation of patients’ dependence in activities of living – Barthel Index [34]

The bilingual questionnaire included both English and Welsh language versions of all the measures. Linguistic validation of the Barthel Index [34] was achieved through adopting the ISPOR guidelines for the translation and cultural adaptation of patient-reported outcome measures [35].

### Discrete choice experiments

Two discrete choice experiments were created using the same experimental design: one for patients (Table 2) and a similar one for carers (Table 3). The final attribute selection was: format of service (group, individual); service provider (hospital stroke team, primary care, voluntary sector); provision of additional social and leisure activities for the patient (provided, not provided); and time to plan and make the journey (1,2,4 hours). Attributes and levels were defined by a review of policy documentation and the two workshops with patients and family carers (n = 8) previously described. Language concordance within healthcare was not included as an attribute in the experiment since this was deemed as a statutory right in Wales.

**Table 2:** Attribute names and descriptions (patient survey)

**Attribute**	**Description**	**Level description (coding)**
Format of services	How help and support is provided for emotional needs, communication problems and physical difficulties	Group support: as part of a group of people who have similar needs (0)
		One-to-one support: on an individual basis (1)
Service provider	Who provides help and support for emotional needs, communication problems and physical difficulties	Hospital stroke team (base)
		Community health team e.g. family doctor, district nurse, therapist in the community (CHT)
		Voluntary organisation e.g. The Stroke Association (VO)
Journey time	Length of time it takes to plan and make the journey to support services	1 hour: to plan and make the journey (60)
		2 hours: to plan and make the journey (120)
		4 hours: to plan and make the journey (240)
Additional social and leisure activities	Social and leisure activities are provided in additional to help and support you receive, that you are able to attend on your own	Not provided (0)
		Provided (1)

**Table 3:** Attribute names and descriptions (family carer survey)

**Attribute**	**Description**	**Level description (coding)**
Format of services	How help and support is provided for your role as a carer for somebody affected by stroke	Group support: as part of a group of people who have similar needs (0)
		One-to-one support: on an individual basis (1)
Service provider	Who provides help and support for your role as a carer for somebody affected by stroke	Hospital stroke team (base)
		Community health team e.g. family doctor, district nurse, therapist in the community (CHT)
		Voluntary organisation e.g. The Stroke Association (VO)
Journey time	Length of time it takes to plan and make the journey to support services	1 hour: to plan and make the journey (60)
		2 hours: to plan and make the journey (120)
		4 hours: to plan and make the journey (240)
Additional social and leisure activities	Social and leisure activities are provided for the person you care for, in additional to help and support you receive, that the person you care for could attend on their own – giving you free time	Not provided (0)
		Provided (1)

#### ***Experimental design***

**Figure 1:**
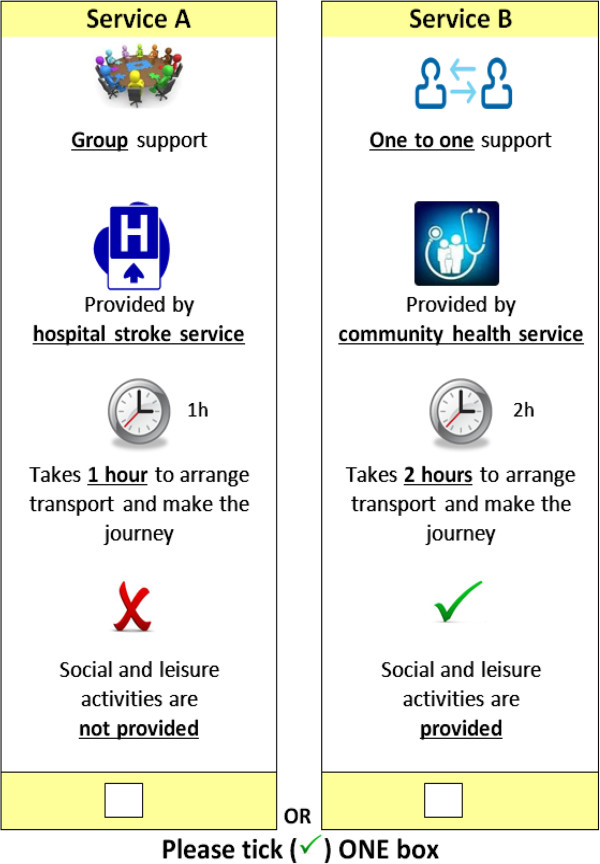
DCE Design.

The DCEs were designed using an orthogonal main-effects plan (OMEP) obtained from a published design catalogue [36]. We used fractional factorial design 44a, folded into nine binary choices, containing four attributes: two with 3-levels, and two with 2-levels (Figure 1). The OMEP ensured orthogonality and level balance [37]. The order of the choice sets in the catalogue was randomised before being transposed into the questionnaire. Dominant choice sets could not be assumed nor removed due to a lack of a-priori evidence on direction of preferences for categorical attributes (e.g. provision of social and leisure activities). The DCE did not contain any additional tests for dominance or transitivity due to the pragmatic limitations of cognitive burden of greater than 9 choices.

##### Methods of data collection

Potential patient participants were sent an expression of interest form which sought clarification of their and their family carer’s willingness to take part. If appropriate, a bilingual study administration pack containing a letter of introduction, information sheet and the relevant version of the survey questionnaire was then sent by post. Where the patient had problems completing the questionnaire independently, completion with the assistance of their family carer was acceptable, and bilingual telephone support for completion was available. Respondents were also able to request to complete the questionnaire with the assistance of a clinical studies officer, in their preferred language. A total of 6 respondents opted to participate in the study in this way. Carers were still able to submit their own survey response. Non-responders received a reminder invitation pack at three weeks and one telephone call to ensure this pack had arrived. Completed questionnaires were mailed back directly to the research team for data inputting.

##### 

**Analysis** A data analysis plan was constructed and agreed prior to data collection, including rules for managing missing and/or incorrectly filled responses. Data were managed in Microsoft Excel (descriptive data) and the STATA Version 10 statistical software (discrete choice data). Random effects logit modelling was used to analyse these data, with choice of service design as the dependent variable [38]. Effects codes were used to enter qualitative discrete choice experiment attributes (format of services; provider of services; and additional social and leisure activities) [26,39]. The β coefficient values derived from the final regression equation were used to estimate the relative importance of each attribute. The significance, sign and magnitude of the coefficient was used to represent the degree of preference for each of the tested attributes. The primary analysis was the marginal rate of substitution (MRS) between journey time and all other attributes i.e. the rate at which a respondent was ready to give up time to plan and make the journey for one attribute level over another. Exploratory analyses of language use; marital status, housing arrangements, age, time since stroke; and distance from nearest hospital were planned. Median splits were used to create two subgroups within an unrestricted model. Log likelihood ratio test of the base case model was compared to the sum of the log likelihood ratio test for the unrestricted model (i.e. random-effects logit regression of sample EQ5D < 0.69 and random-effects logit regression of sample EQ5D ≥ 0.69). The random-effects logit regression only included attributes as covariates. We did not add demographic or clinical data to the models. Patient and carer responses were analysed separately before comparing the results.

## Results

A total of 474 patients were invited to participate in the study, 144 questionnaires were distributed, of which 80 were returned (response rate 46%). Seventy-four family carers expressed an interest via patient invites, 34 questionnaires were returned, also resulting in a response rate of 46%. Two questionnaires were unusable; the preference analysis was therefore restricted to 79 patients and 33 family carers. The characteristics of study participants are described in Table 4. Patient age was significantly lower in those patients who confirmed their interest in the study than those who did not, but there were no significant differences between responders and non-responders to the questionnaire. For family carers, those who cared for older patients were more likely to respond.

**Table 4:** Participant demographics

		**Patient**		**Family carer**	
Demographic		N	Mean (SD)	N	Mean (SD)
Age		80	70.78 (11.12)	34	74.76 (10.00)
Time since stroke (days)		80	331.06 (79.30)	34	338.56 (76.27)
Distance from hospital (miles)		80	15.82 (12.36)	34	18.68 (19.96)
Male N (%)		80	40 (50)	34	12 (35)
Marital status N (%)		77		32	
	Married/civil partnership		48 (60)		26 (81)
	Divorced		6 (8)		2 (6)
	Widowed		20 (25)		0 (0)
	Separated		1 (1)		0 (0)
	Single		2 (3)		4 (13)
Type of stroke N (%)		80			
	Haemorrhage		4 (5)		
	Infarction		75* (94)		
	TIA		1 (1)		
Live alone N (%)		76	21 (26)	32	0 (0)
EQ5D		80	0.63 (0.31)	34	0.80 (0.19)
Barthel		77	86.75 (20.44)	31	69.68 (27.02)**
VAS		74	68.18 (20.14)	32	74.56 (17.32)

*2 went on to have 2nd infarction.

**On behalf of the patient.

On average patients completed 8.3 of the 9 choices and there was no evidence of lexicographic preferences. The format of service, service provider, journey time, and provision of additional social and leisure activities are all important in a patient’s preference for community services after stroke (Table 5), confirming the interpretation of workshop findings. When ranked, the format of the service (group versus one-to-one support) had the most influence on preferences. All attributes had plausible signs in accordance with *a priori* hypotheses (not specified for social and leisure activities).

**Table 5:** Results of the random-effects logit regression model: patient sample (n = 79)

**Attribute**	**Β-coefficient**	**95% CI**		**P value**	**MRS (mins)**	**95% CI**	
Format of service							
_One to one	0.7396*	0.6092	1.0814	0.0000	128.61	87.11	175.91
Service provider_hospital	0.1815*	0.5468	0.3752		31.56	8.52	57.04
_Community health team	0.0474	-0.1074	0.2268	0.5330			
_Voluntary organisation	-0.2289*	-0.4282	-0.1037	0.0020	-39.80	-69.89	-16.00
Journey time (minutes)	-0.0058*	-0.0084	-0.0050	0.0000			
Additional social and leisure							
_Provided	0.3684*	0.2015	0.6638	0.0000	64.07	30.24	103.43
*Constant*	-0.2027	-0.4693	0.0270	0.0630			

No. observations = 653; No. individuals = 79; Wald chi2 (5) = 117.67; Log likelihood = -371.32.

*Statistically significant at P < 0.05.

95% confidence intervals generated using non-parametric bootstrapping.

Marginal rate of substitution values = β-coefficient for significant attribute/β-coefficient for journey time.

The positive coefficients for the attribute of format of service and provision of additional social and leisure activities indicate that patients prefer help and support for emotional needs, communication problems and physical difficulties to be provided on an individual basis; and that they would prefer support services that offered additional social and leisure activities that they are able to attend on their own. The coefficients for service provider indicate that patients would prefer this support to be provided by the hospital stroke team or community health team, but the preference between these two options does not have a statistically significant effect on the patient’s choice. The negative sign on provision by a voluntary organisation, however, means that patients would prefer that a non-statutory organisation did not provide the service. The negative sign on journey time indicates that patients would prefer support services with the shortest length of time taken to plan and make the journey. The MRS indicates the value patients place on each attribute relative to journey time. The results of this analysis suggest that patients would be willing to extend their journey by over two hours for individual rather than group support, and an hour for additional social and leisure services they could attend alone. The negative MRS for provision by a voluntary organisation suggests patients would be willing to forego 40 minutes of journey time to avoid this option.

On average family carers completed 8.6 of the 9 choices and there was no evidence of lexicographic preferences. Only two of the four attributes were statistically significant (p < 0.05): format of service and journey time, with format having the most influence on carer’s preference for support service delivery (Table 6). The signs of both coefficients were plausible, the positive β for format of service indicated that carers would prefer help and support in their role as a carer for someone affected by stroke to be provided individually on a one-to-one basis. The negative sign on journey time indicated that carers would prefer support services with the shortest journey time. MRS analysis showed that carers were willing to extend their journey time by over four and a half hours (274 minutes) for individual rather than group support.

**Table 6:** Results of the random-effects logit regression model: family carer sample (n = 33)

**Attribute**	**Β-coefficient**	**95% CI**		**P value**	**MRS (mins)**	**95% CI**	
Format of service							
_One to one	0.9880*	0.8046	1.5908	0.0000	273.73	160.60	610.12
Service provider_hospital	0.0086	-0.2310	0.2460				
_Community health team	0.0148	-0.2434	0.3099	0.8990			
_Voluntary organisation	-0.0233	-0.3019	0.2115	0.8350			
Journey time (minutes)	-0.0036*	-0.0071	-0.0019	0.0010			
Additional social and leisure							
_Provided	0.2767	-0.0051	0.7050	0.0570			
*Constant*	-0.1865	-0.6162	0.1313	0.2420			

No. observations = 285; No. individuals = 33; Wald chi2 (5) = 49.72; Log likelihood = -163.68.

*Statistically significant at P < 0.05.

95% confidence intervals generated using non-parametric bootstrapping.

Marginal rate of substitution values = β-coefficient for significant attribute/β-coefficient for journey time.

There was no evidence that the random-effects logit model in the base case analysis (restricted model) was statistically different from the unrestricted model (split sample, creating two comparable models) for age, gender, time since stroke, distance from stroke service, and Barthel score. The model that accounted for health utility (p < 0.05) was statistically different, indicating that preferences for support service delivery are influenced by health related quality of life and activities of daily living (Table 7). The service provider was not significant for those with lower health utility compared to higher, who were willing to increase journey time from 76 to 197 minutes to receive one-to-one support and by 26 minutes for additional social and leisure activities. This suggests that health related quality of life is associated with preference for format of service.

**Table 7:** Subgroup analysis by health utility: patient sample

**Attribute**	**Eq5d health utility < 0.69 (n = 39)**							**Eq5d health utility ≥ 0.69 (n = 40)**						
	**Β**	**95% CI**		**P value**	**MRS (mins)**	**95% CI**		**Β**	**95% CI**		**P value**	**MRS (mins)**	**95% CI**	
Format of service														
_One to one	0.9543*	0.7393	1.4900	0.0000	197.06	127.88	336.51	0.5233*	0.2431	1.0217	0	75.52	28.89	130.22
Service provider _HOSPITAL	0.1503	-0.0601	0.3849		n/s			0.2257*	0.0427	0.5280		32.57	5.40	65.12
_Community health team	-0.0272	-0.2704	0.2375	0.8080	n/s			0.1234	-0.0824	0.3940	0.248	n/s		
_Voluntary organisation	-0.1232	-0.4034	0.0987	0.2580	n/s			-0.3491*	-0.7099	-0.2000	0.001	-50.38	-86.41	-22.30
Journey time (minutes)	-0.0048*	-0.0081	-0.0033	0.0000				-0.0069*	-0.0113	-0.0059	0			
Additional social and leisure														
_Provided	0.3858*	0.1137	0.8241	0.0060	79.66	20.91	168.99	0.3715*	0.1274	0.7776	0.008	53.61	13.84	99.18
*Constant*	-0.1424	-0.5636	0.1774	0.3540				-0.2474	-0.7071	0.1181	0.127			
No. observations = 323								No. observations = 330						
No. individuals = 39								No. individuals = 40						
Wald chi2 (5) = 59.09								Wald chi2 (5) = 62.71						
Log likelihood = -179.45								Log likelihood = -185.21856						

*Statistically significant at P < 0.05.

95% confidence intervals generated using non-parametric bootstrapping.

Marginal rate of substitution values = β-coefficient for significant attribute/β-coefficient for journey time.

Service provider base case (Hospital stroke team) calculated by assuming estimate for effects coded omitted variable = -1*(sum of estimated levels).

Table 8 presents the marginal rates of substitution using journey time as the value attribute. The direction of preference was consistent across subgroups for all significant attributes. Carers had the strongest preference for service to be provided in an individual, rather than group format. Older patients would extend journey times by almost an hour to have services provided by hospital stroke teams. Female patients had the strongest aversion to services being provided by voluntary organisations. Additional social and leisure services that patients could attend on their own were valued the most by patients >315 days post-stroke, but did not statistically significantly influence the preferences of carers <315 days post-stroke or carers.

**Table 8:** Summary of MRS by subgroup

		**One-to-one**			**NHS**			**From NHS to CHT**			**From NHS to Vol**			**Social & Leisure**		
	**N**	**MRS**	**95% CI.**		**MRS**	**95% CI.**		**MRS**	**95% CI.**		**MRS**	**95% CI.**		**MRS**	**95% CI.**	
Carers	33	273.73	160.60	610.12												
Patients	79	128.61	87.11	175.91				31.56	8.52	57.04	-39.80	-69.89	-16.00	64.07	30.24	103.43
< 75 years	44	85.34	37.59	139.97							-33.61	-68.35	-3.57	58.49	17.21	106.37
75+ years	35	192.99	124.15	311.92	50.85	8.90	97.95				-50.49	-107.01	-10.15	73.39	18.00	145.38
Female	40	113.74	65.51	170.76	33.78	2.58	67.46				-52.75	-91.13	-21.31	68.08	27.80	115.54
Male	39	156.28	92.55	266.25										63.80	6.07	135.32
< 12 miles	39	113.06	62.34	183.37							-47.77	-92.61	-12.61	79.72	32.50	144.03
12+ miles	40	145.22	85.76	218.41	40.03	8.27	81.51							49.56	2.14	105.41
< 315 days	40	141.54	86.92	216.97							-37.12	-78.10	-3.67			
315+ days	39	113.88	59.42	188.59	36.49	2.28	81.88				-42.52	-90.76	-5.43	95.11	47.82	165.89
Eq5d < 0.69	39	197.06	127.88	336.51										79.66	20.91	168.99
Eq5d 0.69+	40	75.52	28.89	130.22	32.57	5.40	65.12				-50.38	-86.41	-22.30	53.61	13.84	99.18
Barthel < 100	48	150.31	97.33	230.77	41.27	10.34	77.36				-36.96	-78.05	-4.69	67.34	25.30	117.95
Barthel = 100	28	83.64	27.26	156.80							-50.45	-98.17	-12.66	68.41	16.87	133.16

## Discussion

This is the first study to our knowledge that utilises a discrete choice experiment approach to the investigation of stroke survivors’ and family carers’ preferences for community services after stroke. Four attributes that significantly shaped preferences for aspects of the design of community services were identified: the distance people are willing to travel to access services, whether services are delivered individually or within group-based formats, who provides those services, and whether additional support with social and leisure activities is provided. When ranked, the format of the service (group versus one-to-one support) had the most influence on patients’ preferences, followed by access services that included additional support for the resumption of social and leisure activities. Preferences for services provided by voluntary organisations was lowest and was associated with a willingness to forego a reduction in journey time (-40 minutes) to avoid this type of service. Family carers’ preferences were significantly influenced by two attributes: distance to travel and format of the support service provided, and they were willing to travel nearly five hours longer to avoid group-based services. These findings complement more descriptive summaries of a comprehensive matrix of sources of support which may (or may not) exist within regions and localities [8], with which people living with stroke may interact. Specifically, these study findings provide an indication of the relative importance of service components, and point to issues that need to be addressed in community service re-design.

Since this study was commissioned, only one other DCE of stroke patient preferences has reported [21], however, this focused on the in-patient setting, recruiting patients within a few weeks of acute stroke onset, and focusing on preferences for the intensity of rehabilitation provision. Laver et al. [21] found that patients appear to prefer low-intensity rehabilitation programmes, the provision of rest periods and personal rather than computer-facilitated delivery. These findings provide a useful reference frame within which to contextualise a growing evidence-base about the importance of early and intensive rehabilitation [40,41], and specifically a potential mismatch between preference and the development of complex interventions. This mismatch is important to understand within a stroke context, where active engagement between service users and staff is essential for programme success.

Our findings suggest that patients would be willing to extend their journey by over two hours for individual rather than group support. At its simplest level, this finding legitimises the notion of concentrating specialist stroke services, with more localised services provided by generalist and voluntary sector services. This mirrors the development of stroke services within metropolitan areas such as London and Greater Manchester, which have both pursued variations on a ‘hub and spoke’ model of service design [42]. In mixed urban and rural settings, commissioners will still be expected to deliver a comprehensive stroke service, which extends beyond acute care to address life after stroke, and which balances effectiveness and efficiency with the availability of service supports, including human and other resources. This reflects the Care Quality Commission ([43], p16) recommendations that health and social care providers “ensure that their decisions about the future of services are based on a clear understanding of the needs, experiences and priorities of people who have had a stroke and their carers”. Strategic developments within stroke, including the UK Stroke Specific Education Framework [44], provide a structure to help ensure that all staff from across all potential service sectors have the necessary skills and expertise to support life after stroke.

The reorganisation of health and social care services has a number of dimensions beyond effectiveness and capacity, including political and other social and cultural expectations of public services. We have attempted to tap into social acceptability through the degree to which service users would be willing to extend journey times to access services. This is in contrast to the Laver *et al.*[21] study which included willingness to pay, but which is at odds with the public nature of health services in Wales. Study findings indicate that older patients would extend journey time by almost an hour to have services provided by hospital stroke teams, whilst female patients had the strongest aversion to services being provided by voluntary organisations. Whilst demographic factors such as age and gender may affect preferences, these must be balanced with the need for a comprehensive approach within a service model. Nevertheless, this information provides an indication of which groups of service users may (or not) access a service model, and guides activities such as case management that are designed to increase engagement.

The discrete choice experiment data at best demonstrate apathy towards support from the voluntary sector. This is in contrast to very positive reports of experiences of, for example, Stroke Association support, by workshop participants. This for some had been the only source of support after transfer from in-patient stroke services, and the positive evaluation was in keeping with reports that consistently highlight meaningful impacts for patients and families [45]. The discrete choice experiment asked respondents to state preferences between hospital, primary care and voluntary sector provision for support with communication, mobility and emotional issues. Whilst there is little ‘clinical’ evidence to inform decisions about which services are responsible for addressing these issues, it may be that people see different (and complementary) roles for different service providers. Additionally, findings from this study may reflect a lack of personal exposure to voluntary services, which may be viewed as fragile compared to statutory service provision within hospital and community settings. Respondents may also perceive choosing voluntary provision as ‘losing’ NHS provision. It would be sensible to include previous exposure to different types of service providers, and respondents’ perceptions of the quality of these, as covariates in future research.

The findings indicate that services which included attention to social and leisure activities are more important to patients who are more than ten months after stroke (as opposed to earlier). Whilst the desire for support with social and leisure activities is evident, evidence about what this support should comprise is limited. Trials of occupational therapy interventions to promote engagement in these activities have had negative results [46]. The effects of leisure-oriented interventions have been mixed [47,48], perhaps reflecting a limited theoretical base which has focused on awareness raising and education. There is some evidence from a trial of a community-focused rehabilitation programme that, although functional independence may be resistant to change, participation in leisure activity and satisfaction with life are amenable to change [49], possibly through optimising or compensatory strategies [50]. The degree to which these changes are due to enhanced psychological factors facilitating engagement (e.g. confidence/behavioural beliefs), enhanced social support, fewer environmental restrictions to participation, or the renegotiation of leisure activities (or a combination of these factors) is unknown. This study indicates that whilst the development of effective approaches has been limited, the desire for support in this area in the medium to long term is strong, and further research investment in this area is warranted.

The discrete choice experiment approach appears to be a feasible strategy to determine the preferences for patients and family carers. Whilst the sample sizes obtained compare well with those in other published discrete choice experiments, some caution should be taken in assessing the external validity of the results, which also refer to preference at a specific point in recovery from stroke. In addition, and as this study focused on community services, there may be specific issues relating to regional service capacity and population characteristics, such as personal confidence and expectations for stroke and public services in general that mediate preference. Further research is required to test the generalizability of the finding to different stroke populations in different regions, and across different points on the stroke trajectory.

This study involved a relatively homogenous study population in terms of patient characteristics: for example, the median self and proxy rated Barthel Index scores indicated minimal stroke-related disability in the study sample. Further research is required to determine the preferences of sub-groups of patients and family carers in larger and more heterogeneous samples. Finally, further research is required to establish the reliability and validity of discrete choice experiments with this population. People living with stroke may have difficulty with the complexity of completing discrete choice experiment questionnaires, which we were only partly able to manage through our design and data collection processes, and attention to communication needs. However, the mean response of 8.3 choices per patient illustrates in this case patients were willing and able to engage with the activity. Further research may be needed to evaluate different modes of delivery for preference studies for stroke populations with differing needs and socio-demographic characteristics.

## Conclusions

These first insights into preferences for community support services indicate that individual programmes, provided close to home by the hospital stroke team, and which include additional social and leisure activities are important. Patients were willing to extend the time required to plan and journey to services for individual rather than group formats (~2 hours), and additional social and leisure activities (~1 hour), however they would expect ~40 minutes less travelling time if services were provided by a voluntary organisation. Family carers preferred support services provided to them individually, at the shortest journey time. The design of this study, including workshops with service user representatives, and a discrete choice experiment of people living with the consequences of strokes, offers a promising way forward for engagement in the design and development of community services.

## Competing interests

The authors declare that they have no competing interests.

## Authors’ contributions

CB as Principal Investigator, with EF & CP contributed to all aspects of the study including securing funding, study design, data analysis and the interpretation of findings. CB, GWR and HO led the patient and family carer workshops. HO and ER were responsible for participant recruitment and data collection. CB prepared the first draft of the paper, and all authors contributed to its development. All authors read and approved the final manuscript.

## Pre-publication history

The pre-publication history for this paper can be accessed here:

http://www.biomedcentral.com/1472-6963/14/63/prepub

## Supplementary Material

Additional file 1 Thematic analysis of Workshop Data informing the selection of attributes for use in the discrete choice experiment. Click here for file
