# Supplementary material for: Investigating preferences for support with life after stroke: a discrete choice experiment
Source: BMC Health Serv Res. 2014 Feb 8;14:63. doi: 10.1186/1472-6963-14-63 (PMC3929758; doi:10.1186/1472-6963-14-63)
Supplement: Additional file 1 — Thematic analysis of Workshop Data informing the selection of attributes for use in the discrete choice experiment. [file 1472-6963-14-63-S1.docx]

**Additional file 1 -** Thematic analysis of Workshop Data informing the selection of attributes for use in the discrete choice experiment

| **Potential attribute** | **Dimension** | **Exemplar quotations** |
| --- | --- | --- |
| ***Travel to services*** | Convenience | “the convenience side” (Patient EP1; 119) |
|  | Availability | “Availability really isn’t it?” (Patient EP3; 123)  “does ganddo ni ddim bysus, dydyn nhw ddim yn dwad i fyny, cyn belled a lle dwi’n byw” (*We don’t have buses, they don’t come up as far up as where I live*)”. (Patient WP2; 67) |
|  | Accessibility | “We can’t really get in the car very well outside our house because there is traffic passing so it is easier you know…” (Family carer, EP4; 135)  “ond methu dreifio, mae hynny wedi effeithio arna fi yn ofnadwy, a tydi’r gwr ddim yn dreifio, mae o wedi colli ei olwg a wedyn da ni yn hollol ddibynnol ar y plant, neu tacsi.” (But *not being able to drive, that’s had a terrible effect on me, and my husband doesn’t drive, he’s lost his eyesight so we’ve been completely dependent on the children or a taxi*)”. (Patient WP2; 71) |
|  | Ease | “I could come in a car but it’s that much hassle trying to get EP3 in and out outside our house. And when we get there as well”. (Family carer EP4; 139)  “weithiau dydyn nhw [trafnidiaeth cyhoeddus] ddim ar amser cyfleus a mae rhywun yn sefyll yn bob tywydd yn disgwyl, dyna pryd da chi yn sylweddoli mor hawdd oedd medru neidio i gar a mynd a chi eich hun. (*[Public transport] is not always at convenient times so you have to stand about in all weather waiting. That’s when you realise how easy it was to just jump in the car and go places yourself*)”. (Patient WP1; 69) |
|  | Personal meaning of journey | I think it conjures up, well bringing back memories of when you needed the ambulance and it’s the kind of thing you’d rather forget and move on from”. (Family carer EP2; 147)  “er bod ffrindiau a pobl yn dweud ‘mond i chdi ofyn’ dwi’n teimlo dwi’m isio creu trafferth i neb, a dwi ddim yn licio mynd ar ofyn rhywun, da chi’n teimlo bod chi wedi bod mor annibynol a gwneud pethau a da chi yn teimlo bod chi yn creu trafferth i bawb. (*Although friends and people d say ‘you only have to ask’ I feel that I don’t to want to create hassle for anyone I don’t like to ask people, you feel you’ve been so so independent in doing things, you don’t want to create hassle for everyone*)”. (Patient WP1; 105) |
|  | Comfort | “The ambulance was a total rattle trap…. it was worse than a bone-shaker. We had to brace ourselves to stop the seat falling over”. (Patient EP1; 149) |
|  | Time | “Quickness in time as well. The train is probably quicker but by the time you have got to the station and everything; got here from the station the car is still the easiest route” (Family carer, EP2; 131) |
| ***Adaptations at home*** | Time | “We had a stair lift put in, and they were quite quick with that, equipment, like chairs, wheelchairs”. (Family carer EP6; 221). |
| ***Therapy and support services*** | Pro-action | “It was there without having to ask”. (Patient EP1; 237) |
|  | Timing | They were there so he could learn to wash himself and that but he wasn’t ready for that, it was a bit early for him to learn how to wash himself; he didn’t know where he was even. (Family carer EP4; 261) |
|  | Emotional support / feeling cared for | “We just feel that we’ve never been dealt with and forgotten that we are still in the loop even though we don’t need the service as such”. (Family carer EP2; 365).  “I think someone should come and see us, probably every six months. Just to see are you are still living?” (Family carer EP6; 584)  “A dwi’n gwybod dwi wedi bod yn ffodus na ches i ddim stroc fawr, ond taswn i wedi bod yn waeth, hefo’n sefyllfa fi adre, sy’n fregus gyda’r gwr fel mae o hefo’r mab, mi fysa fo wedi codi andros o ofn. Dyna dwi yn deimlo, taswn i wedi bod yn lot gwaeth nag ydw i, fysa gen i ddim y gefnogaeth wedyn dim y dilyniant ynde.”  (And *I know I’ve been lucky that I didn’t have a big stroke, but if I had been worse, with my situation as it is back home, which is fragile with my husband as he is with my son, I would have been very frightened. That’s what I feel, if I had been a lot worse than I am, I wouldn’t have the support then, no follow up, you see*)”. (Patient WP1; 186)  “Ia ond mi ddywedon nhw yn yr ysbyty wrthai y bydde’r Stroke Team yn gyrru amdanai mewn 6-8 wythnos, ond yn yr wythnosau yna, da chi yn teimlo ar goll, mewn ryw limbo rhywsut am nad oedd na ddim byd arall yn mynd ymlaen.” (Yes but *they told me at the hospital that the stroke team would send for me in 6-8 weeks’ time, but during those weeks, you feel lost, stuck in a limbo somehow because nothing else was going on*)”. (Patient WP1; 308)  “Hwne ydi’r peth pwysica dwi’n meddwl yn y broses i gyd- y gefnogaeth emosiynol- achos mae hwne yn cyffwrdd pob agwedd o fywyd rhywun o ddydd i ddydd. Da chi yn teimlo yn ansicr ac yn ofnus ar ol dod adre, oni yn teimlo yn saff yn yr ysbyty ond munud ddois i adre o ni yn teimlo yn ofnus. Er bod y teulu yna, dydyn nhw ddim yn gwir ddallt sut da chi yn teimlo.” (*I think that’s the most important element of the whole process, the emotional support, because that touches on every aspect of everyday life. You’re feeling vulnerable and scared after coming home, I felt safe in the hospital, but the moment I came home I felt scared. Even though the family is there, they don’t really understand how you feel*)”. (Patient WP1; 336) |
|  | Availability | “From what I can gather, every area is a little bit different. Some areas are far better than others”. (Family carer EP6; 395)  “The trouble is there are so many people who have had stroke that need help; like EP5 if she could have a speech therapist by herself so she could go a couple of times a week. There is not a cat in hell’s chance of that happening. There aren’t enough speech therapists, you’d need thousands and thousands because there are thousands and thousands who have had strokes and can’t talk, we’d love a speech therapist but we are never going to get one”. (Family carer EP6; 728) |
|  | Continuity | “One-to-one [therapy] but that happened for a bit about six weeks. Then we had a bit of physiotherapy for a few weeks. Then that finished. Now I give EP5 a bit of physio therapy when she wants me to”. (Family carer EP6; 580)  “We had it [therapy] for what 6 months, then she got pregnant, but she was brilliant. Then when she went off to have a baby, that was it. She said if you want any more help, which he really could do with, then I’m supposed to phone my GP and find out”. (Family carer EP4; 331).  “Like EP3 and the physio, now he knows she isn’t coming he doesn’t bother doing the exercises”. (Family carer EP4, 610)  “wedyn dod adre a ddaru xxxx ddod i weld fi, dwi’n meddwl diwedd Chwefror dwi meddwl oedd hi, a dyma fo yn deud ‘xxxx bach, gyno fi niws drwg i ti’ medda fo, ‘dwi’n cael fy stopio ganddyn nhw’ medda fo. A mae rhywun arall yn cymryd drosodd yr ardal i gyd medda fo, wnawn nhw ddod mewn cysylltiad hefo ti. Does na neb wedi bod,dwi wedi trio ffonio, fatha chi, yn deud bod chi wedi trio ffonio, ond dim byd o sens yna, ond tra roeddwn ni yn ysbytyxxx, mi oedd xxxx yn dod i weld fi dwy-waith bob wythnos yn egluro be oedd yn mynd ymlaen a ‘tisio rhywbeth’ ‘tisio fi neud wbeth’ ‘oes yna rhywbeth alla i neud i helpu’” (Then *when I came home, XXXX came home to see me, at the end of February I think it was, and he said ‘Oh xxxx I’ve got bad news for you, they’re stopping me from coming, and someone else is taking over the whole area’ he said they’ll be in touch. No one has been, I’ve tried phoning, like you said, but no sense what so ever there. But whilst I was in xxx hospital, XXXX came to see me twice a week, explaining what was going on and asking ‘do you want anything, do you want me to do anything, is there anything I can do to help?*)” (Patient WP2; 170)  “mi ddywedodd y meddyg ‘pan ewch chi adre mi fydd...’ Doedd yn siarad i ar y dechre ddim yn dda, a ddywedon nhw ‘fyddwch chi yn gweld y therapydd iaith a’r ffisio.’ Wedyn mi ddois i adre ag o fewn deuddydd mi ddoth y pack o’r [Gymdeithas] Stroc ac mi ddarllenais i hwnnw i gyd. A wedyn ddywedon nhw y bydde pethau yn cychwyn mewn dau dri diwrnod ar ol i mi ddod adre, ond arol 3 mis nes i ddim gweld neb a methu mynd drwadd i neb a wedyn mi ges i, dwi’m yn cofio os na llythyr ne be ges i i gael apwyntiad i fynd at yr adran therapi lleferydd, trwyddyn nhw ges i fynd i’r Art Workshop.” (*The doctor said,* ‘*when you get home there will be…’ My speech at first wasn’t good, and they said ‘you’ll be seeing the speech therapist and the physio.’ And then I came home and within a couple of days, the pack came from the Stroke [Association], and I read that. Then they said that things would start within 2-3 days of being home, but after 3 months, I didn’t see anyone or get through to anybody. Then I got, I don’t remember whether it was a letter or what, an appointment at the speech therapy department, and through them e I went to the art workshop.*)”. (Patient WP1; 174) |
| ***Information provision*** | Mode / source | “Ond tasa rhywun wedi cael ryw gysylltiad hefo rhywun fel y Stroke Assossiation, ella fysa rhywun wedi dallt ag ysgwyddo y problemau, ag ella sa nhw yn dweud ‘o mae hyn yn naturiol, da chi fod i deimlo,’ ond does na neb yna i chi fedru bwrw eich bol a dweud wrthyn nhw, os ydi hyn yn normal.” (But *If we’d had some communication with the Stroke Association, someone may have understood and shouldered the burden , and they may have said ‘Oh! This is natural, you’re supposed to feel like this’. But there’s no one there for you to offload and tell you if this is normal.*)’ (Patient WP1; 248)  “People tell you: don’t look on the internet under stroke, you will frighten the life out of you. Which really you should because you need to know it really. Rather than not know it”. (Family carer EP4; 283)  “I think there should be meetings when they are still in hospital after having a stroke to tell you what is what, and what you are to expect”. (Family carer EP4; 287).  “What I think you should have if there is funding, is a video made up with all the things like you know, what to expect and what the services are. That is what people will do, they will put it on the tele and if they are stuck then they can press their DVD on and got to so and so and it’d come up. I don’t know if that would be expensive”. (Family carer EP4; 886) |
|  | Timing | “I think it was a bit early, it would have been better like now rather than when you are all mixed up and your husband has just come out of hospital and your mind is everywhere, it would be better having it a bit later and a bit more when you have settled down a bit”. (Family carer EP4; 459) |
| ***Mode of support*** | Peer-group support | “hyd yn oed un i un i ddechrau [am gefnogaeth]a gofyn fydde chi yn licio ymuno a grwp lle mae pawb yn gwybod sut da chi yn teimlo” (even *one to one first [for support] and to be asked if you would like to join a group where* )”. (Patient WP1; 343)  “Wedyn wnes i gytuno i fynd [i’r Grwp Gwasanaethau Anadd i’r Ymennydd]. Fydd o yn gyfle i mi gael siarad a gwneud synwyr o’r profiad.” (Then *I agreed to go [to the Brain Injury Service Group]. It will be a chance for me to talk and make sense of my experience*)”. (Patient WP1; 349) |
|  | One-to-one support | “Dwi ddim yn gyfforddus mewn grwp wir, dim bod na ddim yn rong mewn grwp, ond dwi ddim yn teimlo yn gyfforddus. Bydde’n well gen i siarad hefo paned o de, sgwrs dros baned. Neu fyswn i yn teimlo fod rhywun yn dweud bod fi yn siarad gormod neu bod fi yn gwneud lol am hyn a pethau fel na. Fedrai wneud hefo siarad hefo rhywun yn anffurfiol ond dim fel arall.” (*I’m not comfortable in a group really, not that there’s anything wrong with a group, but I don’t feel comfortable. I’d rather have a chat over a cup of tea, otherwise I would feel that someone was saying that I was talking too much or that I was making a fuss about this, that and the other. I could do with speaking to someone informally but not otherwise*)”. (Patient WP2; 345)  “I think with the exercises as well you have got to be very careful, I think you do need a one-to-one thing there, because you can injure yourself if you are not doing it correctly or someone isn’t ready for that”. (Family carer EP2; 902) |
|  | Engagement of family carers | “Now that EP1 is getting better he loves going to his stroke group, and not only does it help EP1 because we are sort of joined at the hip when EP1 was having seizures I wouldn’t leave him at all because you never knew when they were going to happen so we were joined at the hip - where he went I went - and the stroke group gave him two hours off from me, with other people and me two hours off from him to go and root around shops on my own without EP1 being there”. (Family carer EP2; 239)  “The first Saturday of every month, no the second Saturday of every month. They meet in the hospital there. It’s a big meeting, carers in one room and the stroke people in the other, and they have people coming in to talk from various departments again”. (Family carer EP2; 313) |
|  | Access | “We have been approached by a lot of people who have had strokes trying to find out how they can get into the group at [place], they’ve heard about it”. (Patient EP1; 391)  “There are quite a lot [of groups] aren’t there? There are other groups as well but you don’t know where they are. They should send you more leaflets”. (Family carer EP4; 654) |
| ***Support for family carers*** | Stroke patients as carers | “Weithiau mae’n haws siarad a trafod tu allan dwi di ffeindio oherwydd bod y gwr yn sal, mae o mor llawn o’i salwch o, nes weithiau dwi’n teimlo mae’n rhaid i mi gael mynd allan o ty, oni dydd Llun, mi ddoth brawd y gwr a’i wriag acw a mi ddoth cyfnither arall a roedden nhw i gyd yn siarad ac yn trafod a fedrwn i ddim, oedd rhaid i mi godi a mynd. Ond, roedden nhw yn dweud ‘ti di mendio, ti’n edrych yn champion’ ond yn fama(pen) dwi’m yn teimlo yn fel o ni cynt. Mi ddoth y gwr drwadd ‘o odda ni ddim yn gwybod lle oeddech di’ wedyn o ni di mynd drwadd i’r ystafell fwy a cael ryw cry bach tawel, odd rhaid i mi dynnu fy hun o’r peth. Ond swn i yn egluro hynny iddyn nhw, sa nhw ddim yn dallt wedyn be sy’n bod. (*Sometime I have found that it’s easier to talk and discuss things outside, because my husband is ill., He’s so full of his illness that sometimes I need to get out of the house. On Monday, my husband’s brother and his wife came over and another cousin, and they were all talking and discussing and I couldn’t…I had to get up and leave. They were all saying ‘You’re better, you look well’ but inside (in my head) I don’t feel as I was before., My husband came through and said ‘we weren’t sure where you were’ . Then I’d gone through to the living room to have a quiet little cry. I had to remove myself from the situation. But if I’d tried to explain that to them, they wouldn’t understand what was wrong*)”. (Patient WP1; 206) |
|  | Purpose | “I didn’t know what was what and when EP3 came home he was a totally different person, than when he went in. I didn’t know, I hadn’t had any experience in nursing or anything like that so you know I would like to have had a little more information”. (Family carer EP4; 427)  “I think it is helpful to be able to offload sometimes but before you could offload to your wife or husband you have to know that they need to offload and EP1 has never been an open person about what is going on in his head or with his body. It’s difficult for him to admit, if you like, that he is not the person he was”. (Family carer EP2; 690)  EP2: “But do you have support yourself as a carer? when you say that EP5 has care for a few hours can you do the shopping or you go shopping on your own? We can access it if we want it.  EP6: Yes, someone said to me you can get someone for 6 hours.  EP4: Well I do because I feel better knowing that he’s not alone. I wouldn’t trust him, he might do something…  EP6: I don’t want to I haven’t thought about it, I wouldn’t know what to do.  EP4: It is hard when you are forced to go out”. (Family carers EP2,4,6; 802-810) |
| ***Support with return to work and leisure and*** | Leisure related | “Now that he has got this far what we feel is we could use something like they have at [place] where they have the workshops, the afternoon workshops for people after they have had strokes, they do woodwork and mechanics and things like that”. (Family carer EP2; 239)  “roedd ganddyn nhw Art therapy Workshop, ond yn anffodus doedd na ddim digon o gyllid i gynnal 4 sesiwn, wedyn ges i mond mynd i’r 2 olaf, a nes i fwynhau hwnnw yn ofnadwy achos am y dwyawr yna roeddwn ni yn cael switchio ffwrdd a canolbwyntio ar beintio ag o ni’n teimlo bechod na fysa na rhywbeth fel na, mwy o bethau fel yna.” (*They had an art therapy workshop, but unfortunately they didn’t have enough funding to have more than 4 sessions, so I was only able to attend the last 2, and I really enjoyed that because for 2 hours I could switch off and concentrate on painting and I felt it was a shame that there wasn’t more things like that*)”. (Patient WP1; 355)  “doedd o [gweithdy gweithgareddau hamdden] ddim yn canolbwyntio ar unrhyw beth meddygol oedd yn neis” (*It [leisure activity workshop] didn’t concentrate on any medical things which was nice*)”. (Patient WP1; 367) |
|  | Work related | “Being independent, getting yourself back to work or going back to work. The activities really what I was saying about EP1 earlier.  EP1: It is impossible for me, it just can’t happen”. (Mixed EP1-2; 748-752)  “ath fy llygad i yn syth at y pentwr gwaith yna. Fy hun, mae hwnne yn codi ofn arna fi, gan bod fi yn y sefyllfa yna fy hun rwan [yn meddwl am fynd nol I’r gwaith])” (*My eyes went straight for the pile of work on the desk. Personally, that scares me, because I’m in that situation now [thinking of going back to work]*)”. (Patient WP1; 409)  “hefo mynd yn ol i gwaith, dyna oedd yn fy mhoeni fi, meddwl rwan unwaith fyswn i yn mynd trwy drws y swyddfa a dwi nol, wrth gwrs mae pawb o nghwmpas i, ‘o mae xxx yn ol’ straight i fewn i fel o ni cynt, a’n ofn mwy fi ydi, sut ydwi yn mynd i ymdopi gyda hynny.” (*Going back to work, that’s what was worrying me, thinking as soon as I step back in through the office door, people would think , ‘Oh! Xxx is back.’ and that I’d be the same as I was before, and my greatest fear is how I would be able to cope with that*)”. (Patient WP1; 558) |
| ***Follow-up assessment after stroke*** | Experience | “We hadn’t been to the GP in years and years, we hardly knew our GP before we moved here and now we are hardly off his doorstep”. (Family carer EP2; 239) |
|  | Proximity | “We do see [GP] every three months so if you do have any questions”. (Family carer EP2; 564)  “I think the GP is the nearest person and he knows you better than others because of other things you might have gone to him for”. (Family carer EP2; 600) |
|  | Personnel | “He [stroke consultant] is the only person we have dealt with only to do with the stroke rather than general health”. (Family carer EP2; 588)  “Nath doctor teulu ddim neud dim byd, na ddim codi ffon na dim byd, fuodd hi 3 mis cyn fuodd rhaid i’r ferch fynd a fi i’r ysbyty, roedd gen i broblemau gyda’n urine” (*My GP did nothing, didn’t phone or anything. Three months had gone by the time my daughter had to take me to the hospital. I had a problem with my urine* )”. (Patient WP2; 318) |
|  | Responsiveness | “Sa chi yn meddwl basa na rhywun rhwng, i fi, rhwng ybyty xxx, a Dr  xxxx. Rhywun yn fano sa chi yn medru siarad hefo nhw, yn y canol.  Weithiau mae jest cael clust i wrando ar eich pryderon, pethau nad yda  chi yn gallu drafod adre.”  (*You would think that there was someone in between [hospital] and [GP].*  *Someone there in the middle that you could talk to. Sometimes just having*  *someone to listen to your worries, things that you can’t discuss at home*)”.  (Patient WP2; 528) |
| ***Language sensitivity*** | Ease | “Ia mi fydda i yn gofyn ar y ffon, da chi’n siarad Cymraeg, mae’n haws egluro yn Gymraeg, a rwan gan bod fi methu siarad yn iawn, mae’n anodd iawn ar y ffon, a trio gneud o i gyd yn Saesneg, mae’n anodd”  (Yes *I ask over the phone, ‘do you speak Welsh?’ I find it easier to explain things in Welsh, and now because I’m having difficulties with my speech, it’s very difficult over the phone, when you’re trying to do it all in English it’s very difficult*)”. (Patient WP2; 488) |
|  | Emergency | “swn i yn disgwyl bod o yna [ymgynghoriad trwy’r Gymraeg]. O reit, dywed bod angen emergency sydyn bod rhaid cael y meddyg cyntaf yna a hwnnw yn Sais, dyna fo, dim dyna be dwi’n ddeud, dim na dwi’m isio meddyg Saesneg, o na, ond pan tisio egluro i meddyg neu i’r nyrs mae’n haws neud o yn Gymraeg. Dim na dwi’m isio doctor Saesneg” (*I would expect it [clinical encounter in Welsh] to be available. Ok, if there was an emergency and you had to get a doctor and the first doctor doesn’t speak Welsh, well that’s fine, but that’s not what I’m talking about . It’s not that I don’t want an English Dr, but when you’re trying to explain things to the doctor or nurse it’s easier in Welsh. Not that I would refuse and English Doctor*)”. (Patient WP2; 492) |
|  | Understanding | “buaswn i yn dallt gwybodaeth yn well yn Gymraeg nag yn Saesneg” (*I would understand information better if it was in Welsh rather than English*)”. (Patient WP2; 496) |
|  | Therapeutic | “Dwi’n meddwl therapi iaith, ond mae hwnnw yn naturiol o hyd yn Gymraeg” (*Speech therapy I guess, but that’s obviously always in Welsh*)”. (Patient WP1; 502) |
|  | Choice | “Ond tasa fo [darapriaeth gwasanaeth trwy’r Gymraeg] ddim yn bob man, yn sicr dyle bod ydewis ar gael i’r unigolyn.”  (*But if it’s [service provision in the Welsh language] not everywhere the choice certainly should be there for the individual*)”. (Patient WP1; 506) |
